# A Rapid Review of Perinatal Healthcare Experiences of Women With Substance Use Disorders

**DOI:** 10.7759/cureus.75409

**Published:** 2024-12-09

**Authors:** Natasha Stephen, Shelley Doucet, Alison Luke

**Affiliations:** 1 Nursing, University of New Brunswick, Saint John, CAN

**Keywords:** healthcare experience, nurse practitioner, postnatal, postpartum, prenatal, primary care, qualitative, rapid review, substance use disorder, women

## Abstract

The primary aim of this rapid review was to synthesize the literature on the healthcare experiences of women with substance use disorders (SUDs) during the prenatal and postnatal periods. The secondary aim was to discuss the implications of the findings, in the context of primary care nurse practitioner (NP) practice.

A rapid review was conducted, synthesizing research evidence using abbreviated, systematic review methods to generate evidence in a shorter amount of time.

Data sources, MEDLINE (Ovid), CINAHL Full-Text (EBSCO), and PsycINFO (EBSCO), were searched in March 2020. The search strategy was developed for MEDLINE by testing identified terms. Those producing unique results were included in the final search, adapted for each database. Inclusion criteria were primary qualitative studies of prenatal and/or postnatal healthcare experiences from the perspective of women with SUDs, published in English, and available in full-text.

Twenty-five articles were included, the majority from the United States of America. Using thematic analysis, five themes were identified: (i) stigma, (ii) involvement with child protective services, (iii) lack of supportive education, (iv) the impact of relationships, and (v) challenges accessing holistic care.

Further research is required on the prenatal and postpartum healthcare experiences of women with SUDs in the primary care setting receiving care from an NP. More could be learned from the postpartum experiences of women with SUDs, given that the existing literature focuses on pregnancy, with some attention to the neonatal intensive care unit.

Pregnant women with SUDs are over-represented among women who receive suboptimal prenatal and postnatal care. Understanding their healthcare experiences is important for developing strategies to improve access to care, thereby improving health outcomes for both mother and infant. Primary care NPs working with women with SUDs will benefit from this review, to inform their practice when caring for this population.

## Introduction and background

Perinatal substance use remains a global public health concern [1]. Global epidemiologic surveys indicate up to 10% of pregnant women consume alcohol, 3% binge drink during pregnancy, and an estimated 2% of pregnant women report illicit opioid use, including prescription opioids and heroin [1]. These figures likely underestimate the problem, considering the risks and stigma that can accompany revealing prenatal consumption of alcohol and other substances [2,3].

Use of alcohol, illicit drugs, and other psychoactive substances during pregnancy can lead to multiple health and social problems for both mother and child, including miscarriage, stillbirth, low birth weight, prematurity, physical malformations, neurological damage, and neonatal abstinence syndrome (NAS) [4,5]. Infant characteristics associated with NAS, combined with other risk factors in women with substance use disorders (SUDs), may place mothers at higher risk for stress and poor attachment following birth [6]. Dependence on alcohol and other drugs can also impair parental functioning, in turn significantly affecting the physical, mental, and emotional development of children [5]. Although many women quit or reduce substance use once pregnancy is established, others continue to use it throughout their pregnancy, contributing to negative outcomes for both mother and baby [1]. These potential outcomes are compounded by inadequate prenatal care and other complex psychosocial factors [1].

Nurse practitioners (NPs) working in primary care settings are well-positioned to provide prenatal and postnatal care for women with SUDs. NPs need to understand the complexity of the woman’s social, mental, and physical problems to provide appropriate advice and support throughout pregnancy and the postpartum period [5]. A deeper understanding of the prenatal and early postpartum healthcare experiences of women with SUDs will inform the practice of primary care NPs when caring for this vulnerable population. This rapid review will add to the existing literature on SUDs among women in the prenatal and postnatal periods through the synthesis of qualitative research on the subject internationally. This will provide primary care NPs with an important account of available literature to help deepen their understanding of the prenatal and postnatal healthcare experiences of women with SUDs, thereby providing a valuable resource to further inform their practice. 

Background

Women’s prenatal substance use is often driven by factors such as a history of physical or sexual abuse, neglect, trauma, intimate partner violence, mental health concerns, deep poverty, precarious living conditions, child welfare involvement, and physical health problems [2,4]. The responsibility for children is strongly connected to mothers; therefore, mothers with SUDs are looked upon as more deviant than fathers [7]. Furthermore, addiction can make it difficult for women to responsibly care for their children in a way that benefits their development [7]. As defined by the Diagnostic and Statistical Manual of Mental Disorders, Fifth Edition (DSM-5), SUDs are patterns of symptoms resulting from the use of a substance that a person continues to take, despite experiencing problems as a result of their use of the substance [8]. For the purposes of this paper, when referring to substance use and SUDs, this could include any drug and/or alcohol use during the prenatal and postpartum periods that is not recommended by a healthcare provider (HCP). 

Pregnant women who use substances are particularly marginalized as a result of the stigma surrounding their use in pregnancy [9]. Pregnancy may be an opportunity for women, their partners, and other people living in their household to change their patterns of alcohol and other substance use; however, pregnant women who use substances are over-represented among women who receive late, limited, or no prenatal care [9]. It has been shown that traditional prenatal care, based on short appointments and limited psychosocial support, is inadequate for this population [9]. Moreover, women who use substances and receive adequate prenatal care generally have better pregnancy outcomes than women who use substances and do not receive adequate care [10]. Although prenatal care increases favourable outcomes for both mother and child, previous negative interactions with child protection services (CPS) and HCPs are predictors of a lack of routine care during this time [11]. A lack of prenatal care can also limit opportunities for HCPs to offer other health-promoting interventions [10]. 

To develop optimal treatment and promote adherence to critical aspects of maternal and fetal care, it is important to understand the healthcare experiences of women who have SUDs [12]. When designing health and social programs that effectively provide services to this target population, it is critical that women who have SUDs are given the opportunity to provide insight into their needs and experiences [13]. A 2019 meta-summary conducted by Renbarger et al. [13] presents findings from published qualitative studies describing how pregnant and postpartum women with SUD experience healthcare encounters in prenatal, labour and delivery, postpartum, and nursery/neonatal intensive care unit (NICU) settings. Their work provides an important basis for understanding the healthcare encounters experienced by pregnant and postpartum women with SUDs; however, the meta-summary is limited to the United States. 

Using a holistic model of care, NP practice is built on the foundation of a collaborative relationship with the client [14]. Guided by the principles of primary healthcare established by the WHO in 1978 (e.g., accessibility, public participation, health promotion, use of appropriate technology, and intersectoral collaboration), NPs are well prepared to care for and provide support for this population. NPs make what has been traditionally defined as medical practice richer by integrating a nursing-based understanding of the lived experience of patients and families [15]. A better understanding of what is known of the healthcare experiences of women with SUDs in the prenatal and postnatal periods will help inform NP practice to optimally care for this population of mothers and babies. Renbarger et al. state that quality healthcare encounters that overcome barriers and facilitate trusting relationships between pregnant women and their providers are needed to combat the negative effects of substance use during pregnancy [13]. Therefore, an in-depth understanding of the healthcare encounters experienced by pregnant and postpartum women is needed to inform the practice of HCPs involved in their care. 

## Review

Aim

This rapid review explores the healthcare experiences of women with SUDs during the prenatal and postnatal periods. The primary purpose of the review was to synthesize the available literature on the healthcare experiences of women with SUDs during the prenatal and postnatal periods. The secondary purpose was to discuss the implications of the findings in the context of primary care NP practice.

Design

To explore the available literature describing the healthcare experiences of women with SUDs during the prenatal and postnatal periods, a rapid review design was used. This type of review synthesizes findings and uses abbreviated, systematic review methods to generate evidence in a shorter amount of time [16]. Rapid reviews play an important role in informing clinical practice and policy decision-making in a timely fashion, contingent upon the transparency of the reported methodology and limitations [17]. While a rapid review does not include all elements of a systematic review, the limited empirical evidence that compares the two methods suggests that they provide similar results [17]. In addition, rapid reviews are the only review types undertaken within a limited timeframe that assess evidence through the use of a systematic method [17]. Given the seriousness of substance use in pregnancy and the potential long-lasting consequences for the mother and child, this design was chosen to respond to the needs of this population in a timely manner. The systematic process outlined by Tricco et al. for conducting rapid reviews was used, thus minimizing bias and limitations, and strengthening the rapid review (Table 1) [16]. 

**Table 1:** The systematic process applied to the rapid review [16]

Rapid review process stage	Action
Needs assessment, topic selection, and topic refinement	A discussion with the project coordinator for a local parent-child assistance program identified the area of interest. The topic was further refined in partnership with the first author’s master’s in nursing (NP) supervisory committee.
Protocol development	The rapid review team was identified. The research question was developed using the Participants, Phenomena of Interest, and Context (PICo) format. PRISMA was created.
Literature search	MEDLINE (Ovid), CINAHL Full-Text (EBSCO), and PsycINFO (EBSCO) were searched. Keywords included ‘pregnancy, substance use disorder, experiences.’ Limited to English and qualitative literature.
Screening and study selection	2734 articles were screened by a single reviewer by title and abstract using Covidence resulting in 64 articles that were screened by full-text using inclusion/exclusion criteria. A total of 25 articles remained for inclusion.
Data extraction	Data related to women’s healthcare experiences was extracted from the articles.
Risk-of-bias assessment	Inclusion criteria were applied to each remaining article by a single reviewer prior to inclusion in the final draft.
Thematic analysis	Themes were identified across the literature.

Search strategy 

Articles were collected for this review by conducting literary searches of MEDLINE (Ovid), CINAHL Full-Text (EBSCO), and PsycINFO (EBSCO). An initial limited search of MEDLINE and CINAHL was conducted to identify possible search terms and available articles. A librarian (AG) developed a full search strategy for MEDLINE, CINAHL, and PsycINFO by using text words contained in titles and abstracts of relevant articles; index terms used to describe the articles; and a search filter for qualitative research [18]. Identified terms were tested in MEDLINE, and only those producing unique results were included in the final strategy. The search strategy was peer-reviewed by a second librarian (AM) using the Peer Review of Electronic Search Strategies (PRESS) guidelines [18]. The final search strategy was adapted for each database (Appendix A). The search was limited to peer-reviewed articles published in English with no restrictions placed on dates. Inclusion criteria were primary qualitative studies of prenatal and/or postnatal healthcare experiences from the perspective of women with SUDs, published in English, and available in full-text. Studies were excluded if they were from the perspective of other stakeholders, such as spouses or HCPs. Complete inclusion and exclusion criteria are found in Table 2. Search synonyms using the Participants, Phenomena of Interest, and Context (PICo) format are found in Table 3.

**Table 2:** Inclusion and exclusion criteria

Inclusion criteria	Exclusion criteria
Must have a title, abstract, and full text. Must be in English. Must be the experiences of seeking and receiving healthcare from the perspective of pregnant/postpartum women who used/are using substances during their pregnancy and/or during the early postpartum period. Must be peer-reviewed. Primary qualitative research or mixed methods studies that include qualitative methods.	Not from the women’s perspective quantitative research without the inclusion of qualitative methods.

**Table 3:** Search synonyms using PICo format PICo: Participants, Phenomena of Interest, and Context

Participants	Phenomena of interest	Context
Pregnant women	Pregnancy	Prenatal, perinatal, expectant mother, postnatal, postpartum
Substance use disorder	Substance use	Drug use/abuse/addiction, alcoholism/alcoholic, alcohol dependence, alcohol abuse/addiction, alcohol use disorder
Prenatal care	Perinatal care, medical care	Maternal health services, pregnancy complications
Healthcare experience	Patient satisfaction, client satisfaction	-

Search outcome

**Figure 1:**
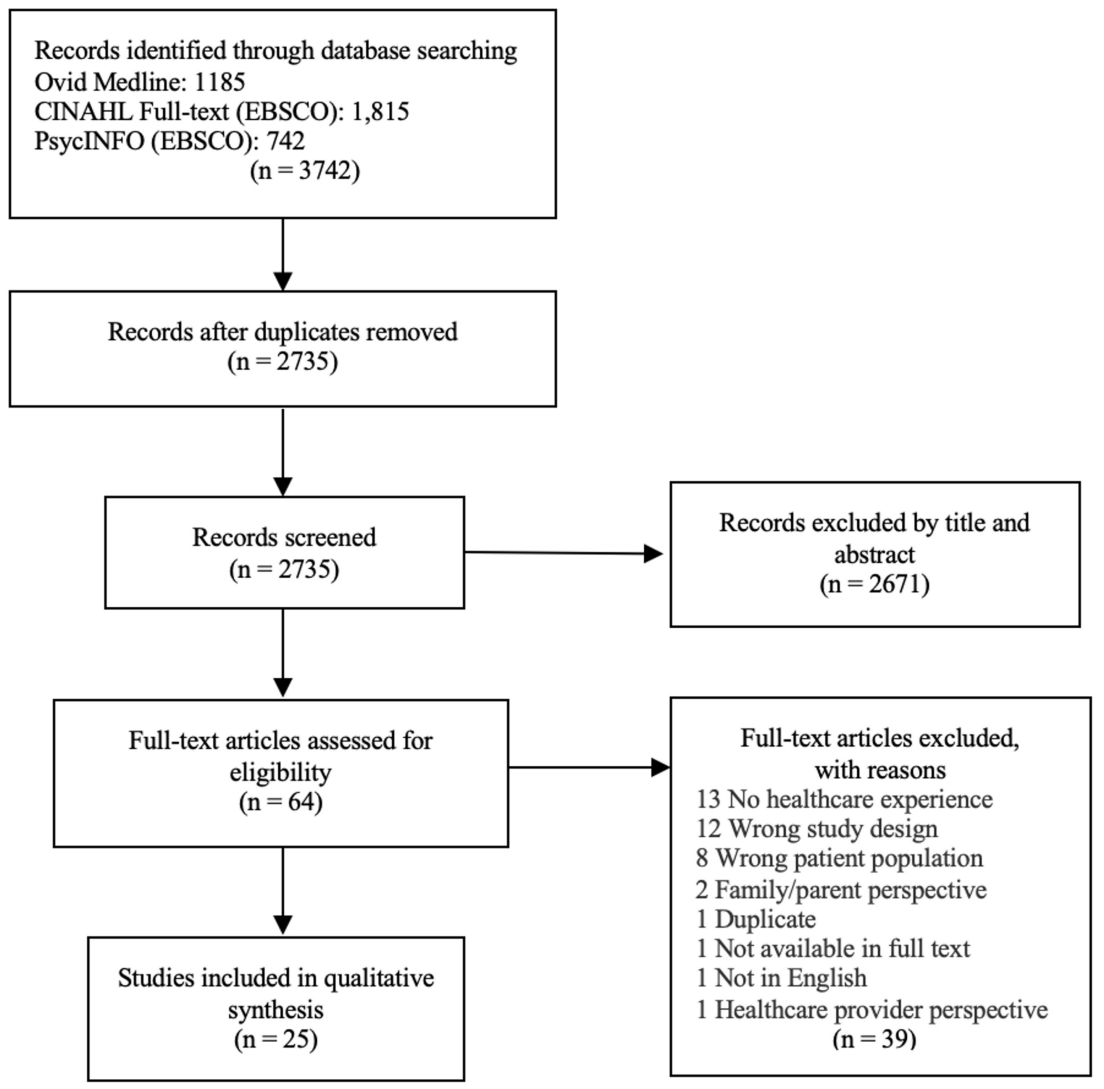
PRISMA flow diagram

A single reviewer can be used in rapid reviews as a means of streamlining the review process [16]. Using the inclusion criteria as outlined in Table 2, the first author screened the citations by title and abstract to determine relevance. This resulted in 64 articles for full-text screening. Again, using the inclusion/exclusion criteria, the first author screened the full-text articles, leaving a total of 25 articles for extraction (Figure 1). 

Quality appraisal

No quality appraisal of studies was undertaken. The purpose of this review was to map the available literature, rather than to evaluate specific effects; therefore, critical appraisal was considered unnecessary [16]. 

Data abstraction

The first author extracted data related to the review question. The data extraction table is included in Table 5 in the Appendices. Extracted data included the author, study aims, population/setting, country of origin, study method, findings, and key study limitations identified by the study authors. 

Synthesis

The six phases of thematic analysis, as outlined by Braun and Clark, were used to synthesize the data [19]. Thematic analysis is a method for identifying, analyzing, and reporting patterns (themes) within data [19]. Throughout the reading of the included 25 articles, any data related to the mother’s experiences with healthcare were copied and pasted into a separate document. During this process, patterns started to develop, and similar excerpts were then grouped together. Once all the relevant data was extracted from the original articles, the development of codes began, which were then developed into themes. 

Results

Twenty-five articles were included in the synthesis. The majority were from the United States of America (14). Other countries included were Canada (four), the United Kingdom (three), Australia (one), New Zealand (one), Slovenia (one), and Sweden (one). All studies were qualitative, and the majority included semi-structured participant interviews and thematic analysis in their design. Five final themes were identified: (i) stigma, (ii) involvement with child protective services, (iii) lack of supportive education, (iv) impact of relationships, and (v) challenges accessing holistic care. 

Stigma

Effects of stigma on prenatal care: Perception of stigma from partners, family, friends, and health professionals had a major impact on the engagement of women with helping services during their pregnancy [11]. This influenced women by limiting their engagement with health services and social supports, including their friends and family [11]. When seeking health care, women emphasized that they wanted to connect with an HCP who would not judge or stigmatize them for their substance use [2,9]. Feeling morally scrutinized deterred them from seeking access to programs they could benefit from during their pregnancy [2]. Many women feared that their drug use had already harmed their baby; some addressed their guilt and concerns by attending prenatal care, while guilt kept others away [10]. Women who attended prenatal care often did so to get reassurance that their babies were healthy, thus relieving stress, anxiety, and guilt [10]. 

Stigma, real or perceived, also contributed to delayed prenatal care and opioid treatment [12]. To avoid detection of drug use during pregnancy, many women delay prenatal care [20]. Attempts to stop using drugs were made before attending prenatal appointments, especially for women with previous CPS involvement [20]. When women proactively sought care, it was because they were committed to acting in the best interest of their children [21].

Effects of stigma on postnatal care: Feeling stigmatized by others, including healthcare staff, left mothers feeling uncomfortable when they visited healthcare settings for postnatal care, such as the NICU [12]. For example, despite attempts to demonstrate maternal capacity by attending to prenatal care and later by caring for their infants in the newborn nursery, women perceived that physicians and nurses in the nursery had negative impressions of them because their infants had NAS [12]. Substance-addicted women described feelings of shame and guilt when they witnessed their infant experiencing withdrawal symptoms [4]. They discussed the inconsolable crying, tremors, and their sense of helplessness in their inability to relieve their infant’s distress [4]. Some did not want to visit the NICU due to these feelings of helplessness [4]. Despite these feelings, most attempted to cope with their feelings of guilt by being there for their infant [4]. Howard noted participants shared similar feelings, including low self-worth, guilt, and shame [12]. Some women in this study believed that the HCPs thought poorly of them as new mothers, affecting their ability to undertake early parenting [12]. Other women expressed feelings of embarrassment, as well as guilt and shame, particularly when they talked about the effect of their substance use on their babies and the rest of their families [22,23].

Cleveland and Bonugli [4] found that some mothers believed that few of the nurses they encountered truly understood addiction, and that this contributed to the way the nurses interacted with them. They wondered if the nurses lacked the necessary education to understand the nuances of addiction and felt the nursing staff could only see the drug use and not the person [4]. Other mothers felt they were deserving of this judgment [4]. Stengel found the women she interviewed presented themselves as responsible mothers committed to reducing risks for their children, but who found the stigmatization of their drug use to be a barrier to accessing the services they needed [21]. 

Involvement With Child Protective Services

Roberts and Pies [10] found that fear of CPS for substance-using women led to different reactions. Most women feared that attending prenatal care while using drugs would lead to CPS reports and the loss of their children [10]. However, a few attended in spite of this fear of CPS because they prioritized their baby’s health, feeling prenatal care would increase their chances of keeping their babies [10]. Work by Chan and Moriarty and Jessup et al. discovered that fear of CPS involvement was a barrier to prenatal care among women with SUDs [11,23]. For some participants in Falletta et al.’s study, CPS was viewed as a path to recovery; some mothers believed the state would care for their children, providing them with the space and time required for their recovery [24]. Further, pregnant women and those with dependent children avoided seeking help for substance abuse due to a fear of losing custody of their children [25]. This led them to develop strategies to conceal their pregnancy, which included isolating themselves, denying their pregnancies, and avoiding medical care [25]. For women who accessed care, their anxiety regarding CPS prevented them from disclosing their entire history of adverse health behaviours to their providers [9,21]. Despite the fear and stress that was found to be common by most, it was also found that child protection concerns were reduced for women if health and child protection professionals had a greater understanding of a woman’s specific concerns and needs [26].

Lack of Supportive Education

More than half of the women in the study by Kramlich et al. described gaps in knowledge and understanding of the impact of substance use on their babies [22], even those with prior experience with substance use and pregnancy seemed unprepared for their babies’ withdrawal symptoms and possible need for pharmacologic therapy [4]. All of the women in the study by Cleveland and Bonugli had family members or significant others with SUDs [4], yet they admitted to having little knowledge of how quickly addiction could occur and the power it could have over their lives [4]. Chan and Moriarty reported that participants received inconsistent information about their condition and its management, including methadone dose safety, neonatal withdrawal, and breastfeeding, resulting in women becoming confused, fearful of outcomes, and more distrustful of health services [11]. 

Mothers also did not feel that they received enough information or support related to opiate exposure with breastfeeding, and its implications for their babies [27]. Postpartum women with SUDs reported that many nurses did not take the time to assist and support breastfeeding, or intentionally sabotaged breastfeeding efforts [28]. Stengel pointed out that women are told during pregnancy of the importance of breastfeeding, but then felt unsupported in doing so once the baby was born. This caused undue anguish and distress to the mother and baby [21]. 

Impact of Relationships

Lack of trusting relationships: Mothers felt that outcomes from healthcare encounters were dependent on their trust in the practitioner; however, traumatic events associated with a previous birth and other healthcare experiences made trust difficult [6,29]. Mothers with infants in the NICU described feeling concerned and vulnerable when they had to leave their babies in the hospital to go home [4]. These mothers tried to stay for as long as possible as a way to cope with this lack of trust [4]. Others experienced a loss of trust in hospital staff when they learned of what they believed to be incorrect information being shared with CPS [4].

Women often fail to trust HCPs and worry that by attending prenatal care, providers would identify their drug use, leading to adverse psychological, social, and legal consequences as a result [20]. Urine tests during pregnancy induced fear, although once women stopped using drugs, some felt urine tests motivated them to stay clean [20]. To help develop trust, women suggested that providers should inform them in advance about urine testing, offering to help right away in response to positive tests, and allowing more than one positive test before reporting to CPS [20]. Rather than disclosing drug use, some women concealed substance use by manipulating drug tests because they felt they could not trust their practitioners [29].

Developing positive relationships: The aspects of care most valued by mothers were non-judgemental attitudes of staff, reassurance, the provision of reliable information, consistency of staff, and high levels of support [23,30]. Gordon et al. learned that establishing good rapport with even one of their practitioners made women feel more positive toward other care providers and more likely to comply effectively with care [28]. 

Mothers entering the NICU for the first time felt relief when they were not judged as they had expected; they discussed how much this meant to them and how it made them feel comfortable when they came to visit their infant [4]. Opportunities to bond with their baby by rooming-in as an alternative to NICU admission had a positive impact on the women’s healthcare experience; they felt this was best for their babies and noted their babies were happier and calmer when they were with them [23].

When HCPs actively involved mothers in the infant’s care, it seemed to help them face the consequences of their substance use [6,23]. When nurses communicated on a personal level, mothers viewed them as caring individuals and described wanting the nurses to provide care for them, as well as their babies [30].

While the role of the father or partner is not a prominent theme in the literature, a study by Milligan et al. considered their involvement to be a key motivator for women using substances during pregnancy in seeking treatment and prenatal care [31]. When a partner was involved, a woman's overall condition improved [31]. Including partners in prenatal care may have many benefits, including improving the prenatal care experience and encouraging stronger families in the future [31].

Challenges in Accessing Holistic Care

Stengel found that women with SUDs struggled to find HCPs that would engage with them [21]. When they did identify someone to work with them, they were often subjected to heightened surveillance that included regular drug testing during their pregnancy [21]. In a study conducted by Salmon et al., the majority of women stated that the support they received was inadequate and that they felt providers focused solely on their medical care and did not include support for substance abuse issues. As a result, issues of substance use were not discussed as part of prenatal care [32]. 

Prioritizing drug use itself over accessing prenatal care was a key barrier for some women [10]. They reported the stress associated with reducing or managing drug use, as well as leaving abusive partners with whom they used drugs, as challenges to seeking care [10]. Some tried to reduce or stop their drug use, while others continued to use it to escape from guilt [10]. In the latter cases, drug use and the associated guilt made it more difficult to attend prenatal care [10].

When seeking treatment, women expressed the value of an experienced HCP, particularly with methadone treatment [33]. Because methadone dosing can be challenging during pregnancy due to hormonal shifts and postpartum depression, it was suggested that working closely with an experienced care provider is critical during this period of treatment [33]. 

Due to the difficulties in finding appropriate care, late detection of pregnancy was a common reason for delayed access to prenatal care [6]. In most cases, pregnancy for women using substances was unintended and came as a surprise [6]. Most were unaware they were expecting until well into the pregnancy, allowing limited preparation time [6].

Women living in rural settings faced waitlists and long travel times for SUD treatment [22]. They also often reported having to visit more than one location to receive prenatal care and substance use treatment due to a lack of coordination between HCPs [22]. Gartner et al. noted a disconnect between support for mothering and support for outpatient addiction recovery, drawing attention to a lack of women-centred, trauma-informed addiction treatment centres where mothers can stay with their families [9].

Discussion

Through the data synthesized in this rapid review, themes were developed to inform our understanding of the healthcare experiences of women with SUDs. These included the effects of stigma on prenatal and postnatal care, involvement with child protective services, lack of supportive education, the impact of relationships, and challenges in accessing holistic care. When recounting their mothering experiences, women with SUDs believed that their pregnancies gave meaning to their lives and provided a window of opportunity for recovery [6,26,29,34]. This knowledge allows HCPs, including primary care NPs, to understand the powerful role motherhood might play as a motivator for pregnant and postpartum women with SUDs to seek treatment [6]. When considering the prenatal and early postpartum care of women with SUDs, HCPs understand that the stakes are high. The included studies demonstrate that this already stigmatized and vulnerable population can become further marginalized as a result of their pregnancy, the majority of which are unplanned [6]. Many women with SUD delay or avoid seeking prenatal care for a variety of reasons, and when they do receive care, it often does not meet their needs. When addiction treatment and prenatal care are substandard, this is of particular concern, given the many deleterious effects that maternal substance use can have on both mother and child [35]. It is also important for HCPs to understand the cyclic nature and associated risk factors of SUDs in women, including past history of childhood abuse, sexual or physical abuse, violence, partner who uses substances, limited family support, homelessness, troubled relationships, unstable family of origin, low self-esteem, and mental health comorbidities [9]. 

Potential Steps for HCPs

Gartner et al. suggest that the disrespectful treatment from HCPs likely stems from their subconscious personal biases and assumptions surrounding drug and alcohol use in pregnancy [9]. The women in this study challenged HCPs to increase their knowledge in this area, encouraging them to acknowledge their perceptions and assumptions about female substance users, especially in pregnancy [9]. These women are equally deserving of high-quality, patient-centred care, and it is necessary for HCPs to understand addiction as a disease to provide non-judgmental care [4,6,9].

Role of Primary Care NPs

The perinatal period is an important time for screening and intervention for factors including social vulnerability and co-occurring disorders, such as SUDs, known to affect pregnancy and infant outcomes [36]. Thoughtful and consistent prenatal primary care can provide opportunities to effectively screen, diagnose, treat, and refer patients to integrated care, improving outcomes for both mothers and newborns [36]. 

Given the extensive training and professional focus of primary care NPs on caring for the whole patient, they are well poised to be the frontline clinicians for this patient population [37]. To address trust building within the therapeutic relationship, Cleveland et al. suggest that interventions that encourage the expression of sensitive and painful feelings are required [6]. Validation of mothers’ concerns and offering support is crucial to the promotion of maternal attachment [6]. Through providing open communication and supportive care throughout the prenatal and postpartum periods, primary care NPs can draw on their holistic approach to healthcare management to promote the development of a strong bond between women with SUDs and their infants, thereby helping to reduce the symptoms of NAS after birth. This is essential, as infants may be a significant motivator for the mother’s treatment [31]. In addition to medical care and pharmacological therapy options, primary care NPs are well prepared to offer nonpharmacologic interventions, including behavioural and mental health treatment, breastfeeding support, and parenting skills [38].

When providing care for pregnant and postpartum women with SUDs, there must be a focus on providing social support to mitigate the stress of parenting brought on by poverty, lack of resources, and health disparities [6]. Howard argues that when treatment providers assume that a pregnant woman with SUD is an unsuitable parent, this allocates blame and responsibility to the mother rather than assessing social resources that would enable safe parenting and recovery [12]. Prior to determining the parental fitness of pregnant or postpartum women with SUDs, the primary care NP must first explore and access available community, social, and healthcare resources to allow for an opportunity to parent under the best possible circumstances. 

Primary care NPs must also understand their patients' personal circumstances so that they know how to support them in attending programs that will benefit both mother and baby. More broadly, with the knowledge of barriers and motivators to care in mind, NPs in primary care can act as informed advocates for identified system changes that can help in the provision of more supportive and comprehensive care for pregnant and postpartum women with SUDs. 

Systems Changes

When considering systems changes to improve healthcare for perinatal women affected by SUDs, the voices of the women in the included studies should be considered. Including women as active participants in their care, improving communication, and developing trust were all key findings. Furthering the development of trusting relationships with HCPs, community outreach, and education could be useful in explaining the role of CPS and the circumstances under which custody of children would be taken [25]. This would help parents understand that they may seek help for SUDs without their children being removed from their care [25]. Improved relationships between CPS and the community may result in more parents seeking care for substance use issues [25].

Chan and Moriarty recommend good team and interdisciplinary communication [11]. Hubberstey et al. found that marginalized pregnant and parenting women who are using substances seek help when health and social services are configured to address their unique realities [2]. Outcomes for mothers and infants improve when accessible, women-centred substance use services are offered in conjunction with prenatal care [2]. Programs that use non-judgemental, relationship-based, trauma-informed, and harm-reduction approaches have been found to be most effective in reaching vulnerable pregnant and parenting women with substance use issues [2,9,25]. Understanding the barriers to accessing such programs is crucial to mitigating them, and understanding motivating factors is important to enhance them [35]. 

Limitations

A rapid review design was chosen as information was required within a short amount of time, and resources were limited. This could be considered a limitation, as a rapid review is not as rigorous as a systematic review. Again, due to time and resources, quality appraisal was not undertaken, and a sole reviewer was used for the study selection and data extraction process. This is in accordance with rapid review protocols, and the method is further strengthened by following the systematic seven‐stage approach outlined by Tricco et al. (Table 1) and working closely with the co-authors throughout all stages of this review [16]. Another limitation is the exclusion of non-English articles, again a result of limited time and resources. The search results included mainly literature from Westernized countries, possibly representing a gap in the literature or perhaps a result of the exclusion of non-English articles. It is important to note that SUD experiences may differ globally, potentially affecting the transferability of the findings internationally. Despite this, there are seven countries represented in this review as summarized in Table 5 in Appendices.

## Conclusions

Considering the interconnected relationship of a mother and her child, they must be considered together by HCPs, including primary care NPs, and the broader healthcare system. This approach is even more important when substance use is involved, due to the many additional risks SUD confers on a child and the mother. NPs and HCPs can work to improve their own knowledge and attitudes regarding this population, while larger systemic changes could offer more inclusive services that incorporate lessons learned from the experiences of the women in this body of literature.

This rapid review demonstrates a need for further research, which aims to investigate the prenatal and early postpartum healthcare experiences of women with SUDs in the primary care setting who are receiving care from an NP. This knowledge could help to better inform the practice approach of NPs when working with this population. Additionally, more could be learned from the postpartum experiences of women with SUD; the existing literature focuses primarily on pregnancy, with some attention to the NICU experience. Further, NPs and other HCPs can act as advocates within the healthcare system and community to improve gaps in pre and postnatal care and substance use treatment for women with SUDs.

## Appendices

Search strategy

Searched on March 10, 2020, results were exported to Endnote and de-duped.

Ovid Medline: 1185

CINAHL Full-text (EBSCO): 1,815

PsycINFO (EBSCO): 742

Total: 3742

Duplicates removed: 1001

Final total: 2741

**Table 4:** Ovid MEDLINE(R) and Epub ahead of print, in-process and other non-indexed citations, daily and Versions(R) 1946 to March 10, 2020

#	Query	Results
1	exp Pregnancy/	882591
2	exp Maternal Health Services/	48175
3	exp Pregnant Women/	7978
4	exp Pregnancy Complications/	419963
5	"pregnan*".ab,ti.	493924
6	((prenatal or perinatal or antenatal or postnatal or postpartum or peripartum or matern*) adj2 care).ab,ti.	30233
7	"expectant mothers".ab,ti.	1003
8	1 or 2 or 3 or 4 or 5 or 6 or 7	1034750
9	exp Substance-Related Disorders/	273884
10	exp Drug Users/	3001
11	((substance or drug or alcohol) adj3 (abuse or dependen* or addict* or disorder or "use" or misuse)).ab,ti.	192100
12	alcoholic.ab,ti.	52541
13	alcoholism.ab,ti.	26687
14	9 or 10 or 11 or 12 or 13	411110
15	((("semi-structured" or semistructured or unstructured or informal or "in-depth" or indepth or "face-to-face" or structured or guide) adj3 (interview* or discussion* or questionnaire*)) or (focus group* or qualitative or ethnograph* or fieldwork or "field work" or "key informant")).ti,ab. or interviews as topic/ or focus groups/ or narration/ or qualitative research/	377936
16	experiences.ab,ti.	192546
17	exp Patient Satisfaction/	87790
18	15 or 16 or 17	596926
18	8 and 14 and 18	1242
19	limit 19 to English language	1185

**Table 5:** Data extraction table *Neonatal abstinence syndrome (NAS); **Neonatal intensive care unit (NICU); ***Substance use disorder (SUD); ****Child protective services (CPS)

Full reference	Aims	Study method	Population/setting	Country	Findings	Limitations identified by author(s)
[11]	Explore the interactions of pregnant women on methadone with helping services and social support, and make recommendations to improve service	Qualitative, semi-structured interviews, grounded theory	Methadone clinic staff, antenatal clinic staff, pregnant women on methadone (n=5)	New Zealand	Few opiate-dependent people have a regular general practitioner and may have perceived or actual barriers to access to care. Perceived stigma deterred these women from engaging with healthcare. Women fear that services will judge and report them.	None identified
[4]	Describe the experiences of mothers of infants with NAS* in the NICU**	Qualitative, semi-structured interviews, thematic analysis	Hispanic, substance-addicted mothers of infants with NAS in NICU (n=15)	United States of America	Participants felt a lack of understanding of addiction among nurses. They felt feelings of guilt and shame when their infants were withdrawing. Participants felt judged by the nurses, interfering with the participants’ ability to trust the nurses.	The sample was all Hispanic and all were in addiction recovery. This may have skewed the findings as these women may have different characteristics than those who are not in a recovery program.
[6]	Describe the mothering experiences of women with SUDs***	Qualitative, semi-structured interviews, thematic analysis	Mothers who had SUD during pregnancy, community-based treatment facility, urban setting (n=15)	United States of America	Identified themes in this study included: Facing the reality of a pregnancy with SUD, finding a higher meaning, dealing with the consequences, managing the details of daily life, and looking toward a future with my children.	Sample lacked ethnic diversity (all Hispanic)
[30]	Describe hospital experiences of mothers who give birth to substance-exposed infants	Secondary analysis, qualitative content analysis	Mothers on methadone treatment with infants admitted to NICU (n=5)	United States of America	Themes identified: try not to judge, score the baby, share with me, and I’m the mother here.	Small sample size (n=5); none of the infants were hospitalized at the time of the interview.
[27]	Describe perceptions surrounding breastfeeding decisions and management among pregnant and postpartum women taking methadone	Qualitative content analysis	Women on methadone treatment who expressed an interest in breastfeeding from an outpatient obstetrics clinic (n=7)	United States of America	Participants experienced guilt about their methadone use and felt that nurses did not want to take the time to assist with breastfeeding or intentionally sabotaged breastfeeding efforts.	None identified
[24]	Understand how pregnant opioid-using women in substance abuse treatment perceive CPS****	Qualitative, semi-structured interviews, thematic analysis	Women currently or recently pregnant with current or recent (past 12 months) SUD at a drug treatment facility (n=16)	United States of America	Fear of CPS can be a barrier to care. Making substance abuse treatment accessible to this population requires recognition of complex feelings toward CPS.	Relatively small sample size, geographically limited area
[25]	Understand the experiences of women with a history of substance dependence when they attempted to gain parenting support	Qualitative, semi-structured interviews, thematic analysis	Women receiving opioid substitution therapy with at least one child less than age 5 from a community outreach program (n=13)	Australia	Participants expressed alienation from mainstream health services. Themes identified included: feelings of guilt, being judged by others, normalizing, and support and learning to be a mother.	Findings are limited to women attending a parenting group and who are enrolled in opioid substitution therapy.
[34]	Explore women's self-reported reasons for pursuing treatment for SUD or hesitating to do so	Qualitative, interviews, thematic analysis	Women in treatment for SUD at a comprehensive care facility for pregnant and parenting women (n=20)	United States of America	Motivators to accessing healthcare and treatment for SUD included: readiness to stop using, concern for the baby’s health, seeking structure, and escaping violence. Barriers to accessing healthcare and treatment for SUD included: fear of loss of custody, judgment or stigma (medical system), and lack of childcare and transportation.	None identified
[9]	To work collaboratively with women accessing an integrated program for women with substance use in pregnancy to learn how services can be improved	Qualitative design, semi-structured focus groups, participatory action framework	Co-researchers were women who had accessed Sheway services (a program for women with substance use during pregnancy) (n=21)	Canada	Identified stereotypes and negative treatment by health care providers as barriers to care. Developing peer-to-peer mentorship was important for support.	Limited time frame for data collection and action planning
[28]	Explore the perspectives of women who have experienced pregnancy and homelessness to ascertain how to improve perinatal care	Qualitative, semi-structured interviews, thematic analysis	Women of diverse parity, most had experienced childhood trauma, grief, mental illness, and substance use. Community setting (n=11)	United Kingdom	Mistrust and fear of child loss to social services influenced interactions with practitioners. Participants experienced stigma from practitioners and concealed their needs, preventing necessary care.	Member checking was not possible to triangulate the findings with the women.
[29]	Understand the experiences of women drug users, seeking and receiving prenatal care and drug services from a specialist clinic	Qualitative, semi-structured interviews, content analysis	Pregnant women using drugs receiving care from a specialist clinic (multidisciplinary care) (n=12)	United Kingdom	Findings included aspects of healthcare received that were most important: high level of prenatal support, pregnancy encouraged them to detoxify, non-judgemental attitude of staff, consistent staff, reliable information, and integrated care.	None identified
[12]	Examine the experiences of opioid-dependent women during their prenatal and early postpartum care	Qualitative, group interviews, interpretive phenomenological analysis	Self-identified postpartum women who used opioids during their pregnancy from community hospitals and treatment centres (n=20)	United States of America	Themes developed around shame and guilt and external stigma. It was recommended interventions involve stigma reduction strategies and increased support for this vulnerable population.	Small sample size, results may not be transferable
[39]	Understand the role of pregnant women with an opioid use disorder participating in medical decision-making regarding their prenatal care while addressing their addiction	Qualitative, group interviews, interpretive phenomenological analysis	Self-identified postpartum women who used opioids during their pregnancy from community hospitals and treatment centres (n=20)	United States of America	Participants reported receiving a wide variety of information and recommendations from various healthcare providers. Many participants also indicated fear of CPS influenced their decision to seek prenatal care.	None identified
[2]	Evaluation of holistic programs serving women at high risk of having an infant with prenatal alcohol or substance exposure	Program evaluation, mixed methods design	Eight programs across Canada were evaluated. Program participants completed in-person interviews at each program site (n=125)	Canada	Women seek help from multi-service programs to: address their substance use, address custody of children, receive help with housing, receive non-judgmental prenatal/health care, and receive peer support.	Voluntary sampling could have resulted in biases and there was a narrow timeframe at each site visit for clients to take part.
[23]	Examine extrinsic barriers to substance abuse treatment among pregnant and parenting women	Qualitative, life history interviews, Mandelbaum analytic framework	Pregnant or parenting women enrolled in perinatal substance abuse treatment program (n=36)	United States of America	Barriers identified included fear of punitive actions from helping institutions. Participants’ desire for child custody and concern for fetal and child well-being was a priority and motivated care seeking despite barriers.	None identified
[22]	Understand the experience of accessing care necessary for substance use disorder recovery, pregnancy, and parenting	Qualitative, ethnography	Rural women with identified SUD who were pregnant or had recently given birth. Large tertiary care hospital servicing rural areas (n=13)	United States of America	Themes identified: challenges of getting treatment and care, opportunities to bond, and importance of relationships (interactions with care providers).	Recruitment was challenging and selection bias may have been present due to the vulnerability of this population. Interviews/observation periods were limited due to the inpatient setting.
[35]	Assess participant perception of an integrated model of care for substance abuse in pregnancy	Qualitative, focus groups	Women who had received addiction and prenatal care at one of two integrated care model sites for substance use in pregnancy (n=19)	Canada	Themes identified: judgement, physician-patient communication, team communication, support groups, and self-responsibility. Women felt more comfortable with providers with non-judgemental attitudes.	Recruitment was difficult and women could not attend due to childcare responsibilities and transportation. Results may not be transferable as participation was voluntary.
[26]	Explore the views of women with opiate dependence on proposed elements for inclusion in a breastfeeding support intervention	Qualitative, think-aloud technique	Opiate-dependent women within 6 months of giving birth, tertiary maternity hospital (n=6)	United Kingdom	Participants were receptive to strategies with a person-centred approach within an emotionally supportive environment. Barriers included discouraging, prescriptive, and judgemental healthcare actions.	Single site and homogeneous sample, limiting transferability of the findings
[33]	Explore perceptions of experiences and challenges with methadone maintenance treatment and obstetrical care among pregnant and postpartum women	Qualitative, focus groups, grounded theory	Pregnant and postpartum women on methadone maintenance at a methadone maintenance clinic (n=14)	United States of America	Participant challenges included finding providers who have experience with methadone maintenance treatment and who are willing to care for pregnant women.	Small sample, single site, women may not have felt comfortable sharing all of their experiences in a focus group setting.
[40]	Understand the experiences of women using illicit drugs during pregnancy, childbirth, and motherhood, and recognize their needs and the obstacles they have to face	Qualitative, semi-structured interviews	Mothers who had used opioids during their pregnancy and labour. All subjects were in addiction treatment programs (n=15)	Slovenia	Most pregnancies were unplanned. Supportive partners/relatives and comprehensive management of pregnancy and drug use had positive effects. Barriers: prejudice and stereotypes from health workers and institutions prevented care.	Small sample
[7]	Identify the experiences of women with SUD who received support at an antenatal and child welfare team and how they perceived the impact	Qualitative, interviews, content analysis	Women who were participating in multidisciplinary services for substance use and prenatal care (n=17)	Sweden	Results showed the importance of creating a non-judgemental attitude to build trusting relationships with the women.	The sample only included women who were receiving services, therefore the experiences of those who were doing less well were not included.
[20]	Understand women’s perspectives or the potential negative ramifications of screening for alcohol and drug use in pregnancy	Qualitative, semi-structured interviews, focus groups	Pregnant or parenting women using alcohol and/or drugs (n=20)	United States of America	Expecting negative consequences from discovered drug use by providers, participants avoided and emotionally disengaged from prenatal care, and attempted to stop using substances that could be detected by urine tests.	Small sample, all participants were receiving substance use services and most had ceased drug use, therefore results may not be transferable.
[10]	Understand how drug use and factors associated with drug use influence women's prenatal care use	Qualitative, semi-structured interviews, focus groups	Women with SUD who were pregnant or had a child less than 2, enrolled in social programs, community-based (n=20).	United States of America	Barriers to prenatal care: drug use itself, fear of CPS, effects of drug use on baby	Small sample, participants were all agency-involved and from one county with few using drugs at the time of participation. Also, many were or had been involved with CPS.
[32]	Explore perceptions of pregnant and parenting substance-using women in an outpatient drug treatment program regarding provider and social support	Qualitative, semi-structured interviews	Pregnant and parenting women who had a history of substance abuse, outpatient drug treatment program (n=20)	United States of America	Most participants were satisfied with social support from family and friends. Many felt that support from medical staff was not adequate. Information from medical providers on the risks of drug use during pregnancy was lacking.	Small sample size and the instrument used was new with no established reliability. Many of the participants were court-mandated to attend the program of study and may not represent the population as a whole.
[21]	Examine the ways in which risk is constructed and managed by those involved in the pregnancy and childbirth of women who use drugs, including the women themselves	Qualitative, semi-structured interviews, inductive thematic approach	Pregnant and parenting women who had used drugs during their pregnancy (n=13)	Canada	Most received late prenatal care (5-6 months), and participants noted that healthcare professionals were unwilling to engage with them if they became aware of their drug use.	None identified
